# Crohn's Disease Imaging: A Review

**DOI:** 10.1155/2012/816920

**Published:** 2012-01-18

**Authors:** Gatta Gianluca, Di Grezia Graziella, Di Mizio Veronica, Landolfi Cinzia, Mansi Luigi, De Sio Ilario, Rotondo Antonio, Grassi Roberto

**Affiliations:** ^1^Radiology Department, Second University of Naples, 80138 Naples, Italy; ^2^San Massimo Hospital, 65017 Penne, Italy; ^3^Nuclear Medicine Department, Second University of Naples, 80138 Naples, Italy; ^4^Gastroenterology Department, Second University of Naples, 80138 Naples, Italy

## Abstract

Crohn's disease is a chronic granulomatous inflammatory disease of the gastrointestinal tract, which can involve almost any segment from the mouth to the anus. Typically, Crohn's lesions attain segmental and asynchronous distribution with varying levels of seriousness, although the sites most frequently involved are the terminal ileum and the proximal colon. A single gold standard for the diagnosis of CD is not available and the diagnosis of CD is confirmed by clinical evaluation and a combination of endoscopic, histological, radiological, and/or biochemical investigations. In recent years, many studies have been performed to investigate the diagnostic potential of less invasive and more patient-friendly imaging modalities in the evaluation of Crohn's disease including conventional enteroclysis, ultrasonography, color-power Doppler, contrast-enhanced ultrasonography, multidetector CT enteroclysis, MRI enteroclysis, and 99mTc-HMPAO-labeled leukocyte scintigraphy. The potential diagnostic role of each imaging modality has to be considered in different clinical degrees of the disease, because there is no single imaging technique that allows a correct diagnosis and may be performed with similar results in every institution. The aim of this paper is to point out the advantages and limitations of the various imaging techniques in patients with suspected or proven Crohn's disease.

## 1. Conventional Enteroclysis (CE)

Conventional enteroclysis is generally performed according to the technique established by Herlinger [1].

A nasoenteric tube is placed beyond the duodenojejunal junction under fluoroscopic guidance. A standard amount of barium (300 mL) and 0.5% methylcellulose solution (1.500 mL) or air, as in our experience, is infused through the nasoenteric tube, achieving optimal double-contrast and small-bowel distention.

Standardized compression views are obtained in all patients for evaluation of the small bowel, especially of the terminal ileum [2].

Crohn's disease has been traditionally investigated with the use of small bowel barium enteroclysis, which detects early mucosal disease (sens. 69.6%, spec. 95.8% [3]) as well as complications such as strictures, fistulae, and abscesses (diagnostic accuracy 80.3%) [3, 4].

**Figure 1:**
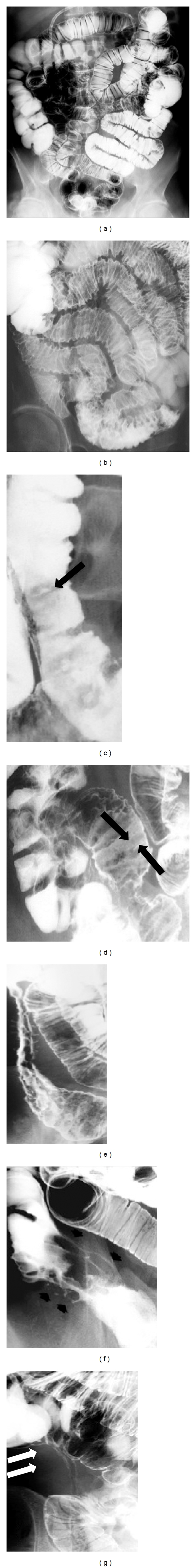
Conventional enteroclysis. (a) Conventional enteroclysis: panoramic view. (b) Mild wall thickening in the ileum. (c) Scattered linear aphthoid lesions (arrow) in a segment of distal small bowel. (d) Mucosal ulcers (arrows). (e) Typical cobblestone-like nodular filling defects and ulceration. (f) Fistula (arrows). (g) Stenotic loop (arrows).

Radiologic findings include irregular thickening and distortion of the valvulae conniventes, loops adhesions (mass-like effect), or separated loops because of wall thickening and mesenteric inflammatory infiltration [5] (Figure 1).

Transverse and longitudinal distribution of ulcerations can separate islands of thickened internal wall, resulting in the typical cobblestone appearance.

Strictures are often separated by healthy bowel tracts (skip lesions); impaired small bowel peristalsis is commonly observed within rigid stenotic tracts. Extrinsic compression may be observed, due to mesentery lymph node enlargement [6]. In partial obstructing stenosis, enteroclysis may provide higher sensitivity than enterography for detection of lesions in the small bowel [7].

Although it manages to accurately detect the location and extension of Crohn's disease (sens. 98%, spec. 97% [8]), it is unable to provide information on extraluminal lesions [9], and capsule endoscopy and double balloon enteroscopy have replaced the enteroclysis as gold standard technique, even in clinical practice [7].

However, barium enteroclysis may be required as an additional test in a small group of patients with a high clinical suspicion but a negative CT study [10].

## 2. Ultrasonography (US)

Ultrasonography is an accurate, noninvasive, painless diagnostic tool with the capability of being used extensively in the clinical setting.

The examination consist of a global evaluation of the small bowel and colon with standard resolution US (3.5–5 MHz), followed by a focused high-resolution study (7–12 MHz). B-mode US may visualize five concentric layers with different echogenicities [11].

 The first layer is the echogenic interface, followed by a hypoechoic mucosa, an echogenic submucosa, a hypoechoic muscular layer, and a echogenic interface between the serosa and the adjacent fatty mesentery [12].

Each layer does not correspond exactly to a defined histologic layer but rather an interface between adjacent layers [13].

The normal thickness of the small bowel is comprised ≤3 in the distended bowel and ≤5 mm in the nondistended bowel.

**Figure 2:**
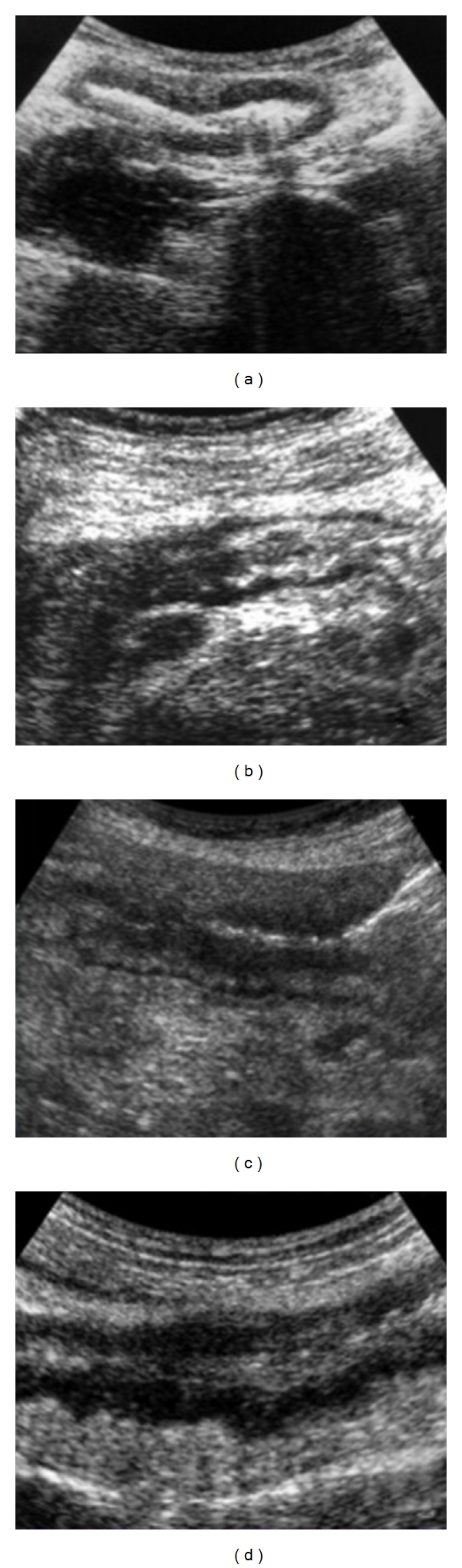
US. (a) Wall thickening without stratification and hyperecoic lumen. Regular outer margin of the loop. Mesenteric fat hypertrophy. (b) Coexistence of two patterns. Stenotic and thickened loop with preserved stratification and an adjcent segment with loss of stratification. (c) Stenotic intestinal tract characterized by marked dilatation of the bowel lumen, with thickened bowel wall. (d) Wall thickening with loss of normal stratification. Discontinuous outer margin with hypoecoic indented irregularities due to extramural findings.

Intestinal US allows the visualization of wall thickening of the relevant loop, with the loss of normal stratification and motility, lack of compressibility by the transducer, narrowing of the lumen, conglomeration, the possible coexistence of mesenteric thickening, increased lymph nodes, abdominal fluid and abscess, fistulas and stenoses related to dilations of the upper loops [7, 11] (Figure 2).

However, the accuracy of US is highly dependent on factors such as experience level of examining physician and location and severity of the disease [7].

When sensitivity is estimated based on disease location, the highest values are found for anatomic areas easily accessible by US, such as terminal ileum and left colon, whereas the diagnostic accuracy is lower for upper small bowel and rectum [14].

Ultrasonography has the further disadvantage of being difficult to perform on overweight patients affected by severe meteorism. Furthermore, an ultrasound exam does not allow operators to detect superficial intramural lesions [15–17].

The significant heterogeneity of the estimates of diagnostic accuracy (sensitivity 75–94% and specificity 67–100% [18]; sensitivity and specificity 90% and 96% [19]; sensitivity and specificity 85% and 98% [7]; sens. 92% and spec. 97% [8]) precluded the possibility of obtaining a cumulative value of diagnostic accuracy.

In general, the magnitude of US changes has a high correlation with endoscopic and histological magnitude of alterations and a weak correlation with indexes of clinical activity and biomarkers [7], especially in fistulae (sens. 71.4%, spec. 95.8%, acc. 85.2%) and abscesses (acc. 88.5%) evaluation [3].

Moreover, the bowel hydrosonography (with oral nonabsorbable solution) results in an increase in the sensitivity of US for the detection of segments with active disease [20].

To date, sonography of intestinal loops is useful as the preliminary examination [21], can help in clinical diagnostic confidence, and can provide important diagnostic findings, suggesting the use of the other imaging technique. The use of US has also been proposed in the followup of patients with known Crohn's disease in asymptomatic patients in order to identify the occurrence of complications at an earlier stage [22] and recurrences (diagnostic accuracy 72.7%) [23].

All studies showed a high accuracy of US for the diagnosis of postsurgical recurrence in CD, detecting almost all cases of severe or complicated recurrence, as well as high sensitivity and specificity in differentiating mild from severe recurrence, especially after giving oral contrast [7].

US has demonstrated a high diagnostic accuracy for the detection of small bowel fistulae, abscesses, and stenosis [24].

For the detection of internal fistulas, the combination of small bowel enteroclysis and US significantly improved diagnostic accuracy (small bowel enteroclysis 84%, US 85%, combination 91%) [7].

In conclusion, in known Crohn's disease for following disease course and evaluating relapses and extramural manifestations, US is an excellent tool (sens. 88.4%, 93.3%, acc. 90.4%) [25].

## 3. Color-Power Doppler (PD)

Color and power Doppler US permits the measurement of arterial and venous flows in the upper mesenteric vessels, the evaluation of the increase of the relevant loop, determination of alterations in the vascular and microvascular nature of the inflammatory process and association with neoangiogenesis in the intestinal wall [26].

Color and power Doppler imaging usually is performed with parameters optimized to detect low velocity and low-flow states (pulse repetition frequencies 800–1500 Hz, wall filter 40–50 Hz, maximal color signal gain immediately below the noise threshold, high levels of color versus echo priority, and color persistence) [13].

The intensity of the vascularity may be subjectively categorized as mild (small focal area of color signal), moderate (multiples areas of weak color signal), or marked (multiple areas of color signal) because some studies have found that increased vascularity of the diseased bowel wall correlates with the activity of the disease [26].

Dopple, sonographic parameters of superior mesenteric artery are significantly correlated with disease activity in nonoperated and noncomplicated Crohn's disease [27].

Combination of B-mode and power Doppler sonography has a high accuracy in the determination of disease activity in Crohn's disease when compared to ileocolonoscopy [27].

**Figure 3:**
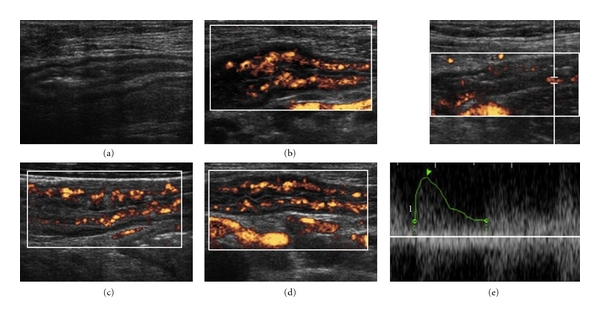
PDs. Wall thickening in B mode (a), in PD (b), (c), (d), and arterial doppler spectrum (e).

To date, the use of power Doppler US has been suggested to improve the diagnostic accuracy of US, particularly in discriminating inflammatory from fibrotic strictures, in better defining the presence of internal fistulas, and to differentiate these lesions from intra-abdominal abscesses [13, 15–19, 21, 22, 28] (Figure 3).

## 4. Contrast-Enhanced Ultrasonography (CEUS)

The limit of US evaluation is the impossibility of assessing bowel wall vascularization and the differentiation between thickening due to active inflammation or fibrosis cannot be reliably made with ultrasound. However, the bowel wall neovascularization is an early pathological change occurring in patients with active CD [29].

The availability of dedicated contrast-specific techniques overcomes the limitations of CD-US with microbubble contrast agents, including blooming artifacts and the limited visibility of vessels with a slowflow [13] and has enabled ultrasonography to obtain information regarding the perfusion behavior of the organs and their diffuse or focal diseases [30].

Microbubble contrast agents are mainly blood-pool agents and present a pure intravascular distribution and allow to increase the backscatter signal from blood cells. Microbubbles consist of small gas particles with a diameter of 2–6 *μ*m with a stiff or flexible shell composed by biocompatible materials (proteins, lipids, or biopolymers) presenting an overall thickness from 10 to 200 nm.

Insonation techniques are available for CEUS. The high-transmit-power insonation produces extensive microbubble destruction with the production of a wide-band irregular harmonic signal. Low-transmit-power insonation (about 30–70 KPa) produces microbubble resonance with production of regular harmonic frequencies and allows real-time scanning, and it is the technique of insonation which is usually in the clinical practice.

Now are available also specialized contrast-specific US techniques, such as pulse inversion, recognition imaging, power modulation, and contrast pulse sequence [31].

**Figure 4:**
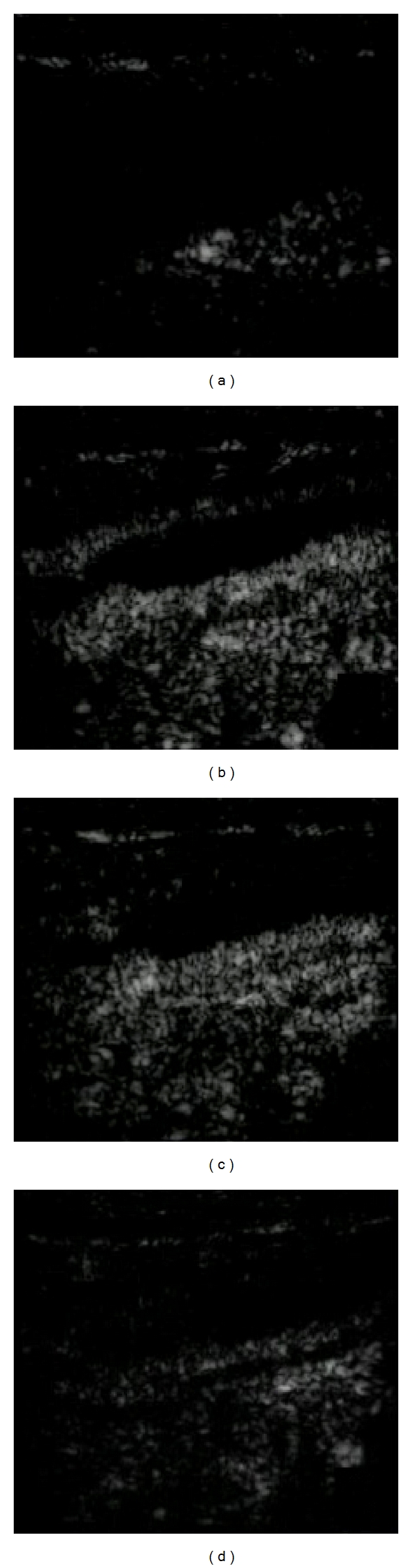
CEUS. Last ileal loop wall thickening and submucosal contrast enhancement after contrast medium (SonoVue, Bracco). 0 (a), 15 (b), 30 (c), and 45 (d) sec.

CEUS provides an adequate evaluation of the increased parietal vascularization in the active phase of the Crohn disease. It might help in characterizing bowel-wall thickening by differentiating inflammatory vascularization, edema, and fibrosis and may help to grade disease activity by assessing the presence and distribution of vascular perfusion within the layers of the bowel wall, although it is limited to the evaluation of a specific loop (Figure 4).

Four different perfusion patterns of bowel enhancement related to Crohns activity have been recently proposed: (a) a complete enhancement of the entire wall section, from the mucosal to the serosal layer; (b) the absence of enhancement only in the outer border of the muscularis propria; (c) the absence of enhancement both in the outer and in the inner border of the bowel wall and enhancement only in the intermediate layer; (d) the complete absence of enhancement in the entire wall section [29].

Contrast-enhanced US could classify severity significantly better than Doppler-US signal and measurement of mural thickening (*P* < 0.001) [32].

Patients with Crohn's disease require frequently multiple imaging examinations. CEUS is a noninvasive technique, which is also more comfortable for the patient with significant diagnostic accuracy. The high sensitivity and temporal resolution of CEUS in the assessment of small bowel vascularity is the real strength of this technique [30].

CEUS can become the most useful imaging modality in the differential diagnosis between fibrotic and inflammatory thickening, in the detection of possible disease complications (abscess, phlegmons, and fistulas) and for assessing the efficacy of medical therapy in reducing bowel-wall vascularity in patients with chronic inflammatory disease.

To date, one study evaluated the accuracy of contrast-enhanced US for assessment of activity in CD, showing that the technique has a high sensitivity and specificity (93% and 94%, resp.) [33], (sens. 81%, spec. 63% for semiquantitative method; sens. 81%, spec. 55.6% for quantitative method) [29].

The comparison of the diagnostic accuracy of conventional US, Doppler US, and contrast-enhanced US for assessment of disease activity showed that the sensitivity of three modalities of examination and specificity are virtually identical (94, 94, 94% and 97, 97, 97%, resp.) [33].

 In conclusion, CEUS has a high sensitivity and specificity in detecting inflammatory activity and a strong correlation with the CDAI (sens. 93.5%, spec. 93.7%, acc. 93.6%, correlation coefficient 0.74; *P* < 0.0001) [33].

CEUS allows real-time assessment of the bowel-wall perfusion with the highest temporal resolution of all imaging techniques and with a spatial and contrast resolution that rivals that of CT and MRI. In consideration of the need for patient comfort, especially in pediatric imaging, CEUS might become the most useful modality for assessing the efficacy of medical therapy with chronic inflammatory disease.

The routine use of the CEUS in the clinical assessment of the patient with active Crohn's disease for therapeutical and surgical management should be suggested [33].

## 5. Multidetector CT Enteroclysis (MDCT-E)

Multidetector CT enteroclysis was introduced as an alternative imaging method to overcome the individual deficiencies of CT and conventional enteroclysis and to combine the advantages of both in one technique.

**Figure 5:**
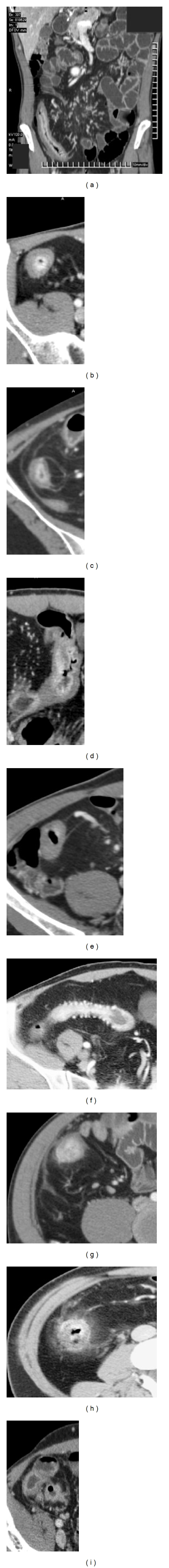
MDCT-E. *Intraintestinal findings*: intramural (a), (b) wall thickening (b) (“double halo sign”), (c) hyperemia of the mucosa, (d) ulcer, (e) stenosis, extramural (f) engorgement of vasa recta (“comb sign”). *Extraintestinal findings*: (g), (h) lymph nodes involvement and mesenteric fat stranding, (i) abscess.

MDCT-E has been described as highly accurate in revealing mural and extraluminal manifestations of disease, including abscesses, while conventional enteroclysis was superior for luminal abnormalities and ulceration (Figure 5) [34, 35].

CT enteroclysis can be performed by using positive enteral contrast material without intravenous contrast material or neutral enteral contrast material with intravenous contrast material [36].

The advantages of neutral CM through the lumen outweigh those offered by positive CM for the following reasons: lower costs, low viscosity, faster injections, and better view of enhancement, wall thickening, and mesenteric involvement. Positive CM through the lumen is useful in the case of contraindications to CM intravenously injected. As far as our study is concerned, we combined neutral and intra-venously injected CM through the lumen in all patients [37].

In our experience, before the exam, patients take laxatives for small-bowel and colon cleansing. Then a nasoenteric tube—150 cm long, 21 mm in diameter, 2.8 mm in external diameter, and a distal end closed by a plastic tip with 4 side holes [Guerbet, Paris]—is placed. The patient is moved to the CT room where scout-view and volumetric scan are carried out.

A layer not wider than 3 mm and a reconstructing interval not larger than 5 mm are chosen.

20 mg of hyoscine butylbromide are administered intravenously in order to reduce intestinal peristalsis and segmentation of the intestinal loops and foster their distension.

1800 mL of water at temperature 37°C is used as a neutral contrast agent and administered through a peristaltic pump so as to obtain a suitable distension of the intestinal loops.

The initial 500 mL is flushed through the tube at a speed of 120 mL/min in order to avoid stress caused by sudden loosening. Then the inoculation of another 1000 mL at 240 mL/min follows, with the aim of loosening the loops and pushing the contrast medium (CM) forward. The last 300 mL is injected at 120 mL/s.

After introducing 1500 mL of intraluminal contrast medium, iodinated contrast agent is injected intravenously, 1 mg iodine/kg body weight (BW) through a mechanic injector at a concentration of 400 mg iodine/mL (“Iomeprol” and ‘“Iomeron 400”' Bracco, Italy) with a 80 s delay in scanning. In the first 40 s, the CM is injected at a speed of 1 mL/s, whereas in the remaining 30 s it is administered at a speed of 3 mL/s. The exam is carried out with one volumetric acquisition at 70 s during breathing-in apnea, thus reducing the CT-dependent patient's dose. CT images are analyzed on a soft-tissue window (30-HUcenter level, 400-HU-window width).

Multiplanar reconstructions are undertaken in all patients to help interpret conflicting findings in axial scans, improve the detection of lesions, and increase the capability for assessing lesions' extension.

Patients with the disease do not form a clinically homogeneous group, and they may be very different from one another, with different clinical situations influenced by individual expression of the disease and possible previous surgical procedures; this does not always allow a uniform and reproducible clinical and radiological standardization of the disease [38, 39].

In patients suspected of having Crohns disease, MDCT-E is accurate in depicting mucosal abnormalities, bowel thickening, mucosa hyperemia, ulcers, stenosis, engorgement of vasa recta, and lymph nodes and mesenteric involvement [40–42].

It is superior to CT enterography in that it provides a suitable uniform distension of the lumen, thus allowing assessment of wall thickness [43].

Evidence suggest a high sensitivity, specificity, and accuracy in the evaluation of relapse of ileocolic anastomosis [7].

It also has higher sensitivity and greater interobserver reliability if compared to MR enteroclysis and does not entail any risk of capsule retention to the patient while performing video capsule endoscopy [44].

Nonetheless, MDCT-E has its drawbacks. Take for instance, ionizing radiation, time needed for placing the nasoenteric tube, high costs, contrast medium intravenously injected, likely inhalation of contrast medium injected into the lumen, necessity to attain a suitable distension of the lumen, and lower sensitivity when it comes to identifying lesions of the mucosa and jejunum.

Differences in specificity and sensitivity can be ascribed to a distinct lack of standard protocols [45–49] and to the different methods followed for data analysis (CD diagnosis sens. 84%, spec. 95% [19]; location and extension sens. 88%, spec. 88% [7]; disease activity and severity sens. 81%, spec. 88% [7] or sens 89%, spec 80% [50]; complication sens. 81%, spec. 98% [7]; extraintestinal complications sens. 100% [50]).

Moreover, for the identification of abscesses, accuracy is higher for CT (92%) than for US (87%) because false positive results in US studies [7] and significant correlations are observed between the intensity of various CT changes and the severity of endoscopic lesions [51].

For these reasons, by defining universal procedures (e.g., patient's preparation, performing techniques, diagnostic standards for IBD, etc.) we will be able to increase sensitivity and specificity values in addition to being able to establish a more accurate diagnosis in order to establish therapy programs which suit the individual's needs [37].

To date MDCT-E is indicated in case of patient with initial diagnosis of Crohn's disease, suspected complications or recurrence [39].

## 6. MRI Enteroclysis (MR-E)

MRI enteroclysis is a noninvasive, nonionizing radiation diagnostic technique able to obtain multiplanar diagnostic information about intra- and extraintestinal lesions and evaluate disease activity [43].

The high soft-tissue contrast, multiplanar capabilities and possibility of obtaining functional information make MR imaging the ideal technique for evaluating small-bowel inflammatory disease (sens. 78%, spec. 85% [7]; sens. 93%, spec. 93% [19]).

In addition, MR imaging has the advantage over traditional techniques of visualizing the entire thickness of the bowel wall and the perivisceral loose connective tissue [53–55].

Some technical aspects, in particular distension of the bowel and use of a luminal contrast, may affect the accuracy of MRI for assessing changes associated with active disease such as wall thickening and enhancement of bowel wall after MRI contrast administration [56].

However, the study protocol has not yet been standardized, and some controversy remains regarding the value of nasoenteric intubation [57].

Some authors [58] suggest that although bowel distension is greater in patients undergoing MR enteroclysis than in patients undergoing MR enterography, this produces no significant difference between the two groups.

**Figure 6:**
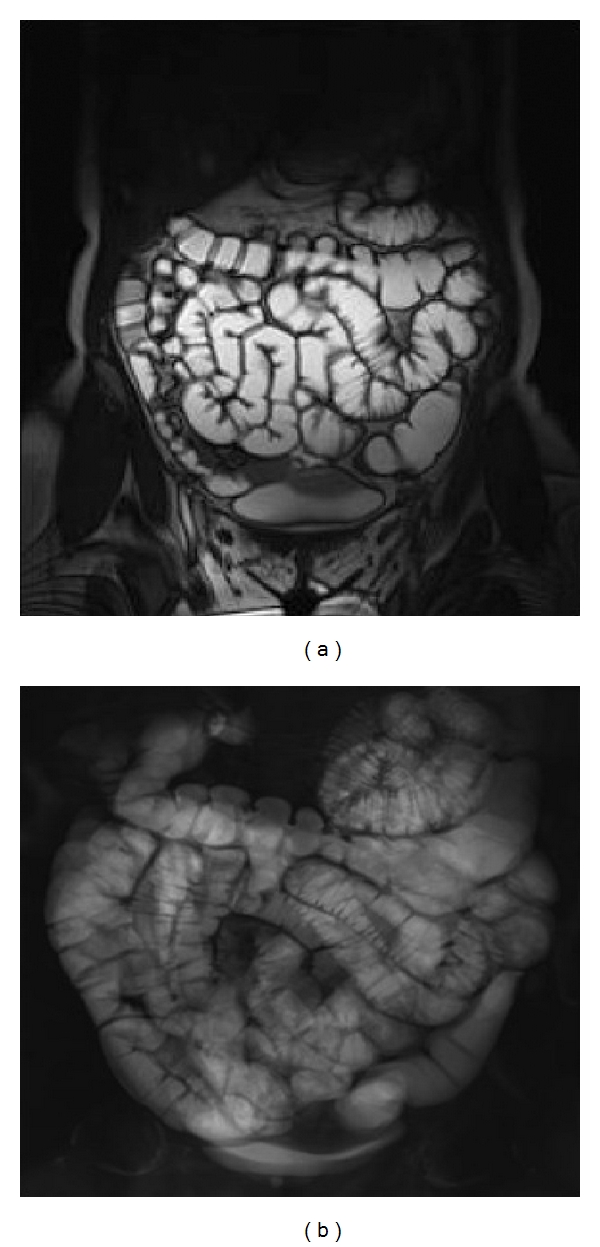
MR-E. (a), (b) Coronal true fast induction steady-state potential and single-shot hydrographic sequence showing a suitable degree of jejunum distension.

Nevertheless, MR enteroclysis (sens. 90%, spec. 100%) proved to be more effective than MR enterography (sens 89%, spec 67%) in evaluating stenosis and its significance and also in evaluating bowel-wall thickness (Figure 6) [59], and examination with the use of peroral contrast agent administration may not be accurate for detection of early mucosal lesions of CD [50].

Moreover, this method is less accurate in the detection of a small-bowel stricture, especially a partially obstructing stricture [50].

In our experience, two days prior to the examination, all patients take a 2,000 cc solution of polyethylene glycol (PEG) in water (SELG 2000, Milan, Italy) to cleanse the bowel and are invited to follow a semiliquid diet and on the day of the examination to have nothing by mouth. Allergic patients undergo desensitisation therapy with cortisone and antihistamines for 3 days prior to MR examination [60].

Distension of the small bowel is obtained with the administration of an 1,800 cc solution of PEG in water (SELG 2000). The solution is injected via the nasoenteric tube after they have entered the scanner (MR enteroclysis).

Patients are premedicated with 20 mg i.v. of hyoscine N-butylbromide to reduce intestinal peristalsis and segmentation of the bowel loops during the examination and are imaged in the prone position with a 1.5-T MR scanner (Magnetom Symphony, Siemens, Germany) and phased-array body coils.

**Figure 7:**
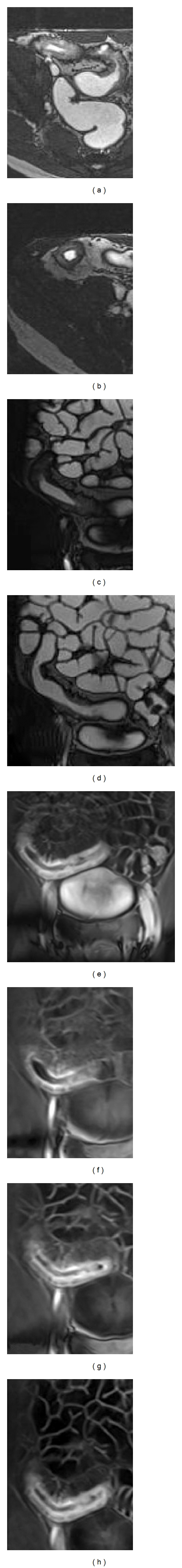
MR-E. Last ileal loop wall thickening (a), (b) axial TRUFI T2. (c), (d) Coronal TRUFI T2. (e) Coronal FLASH 3D. (f), (g), (h) Coronal FLASH 3D postcontrast medium.

**Figure 8:**
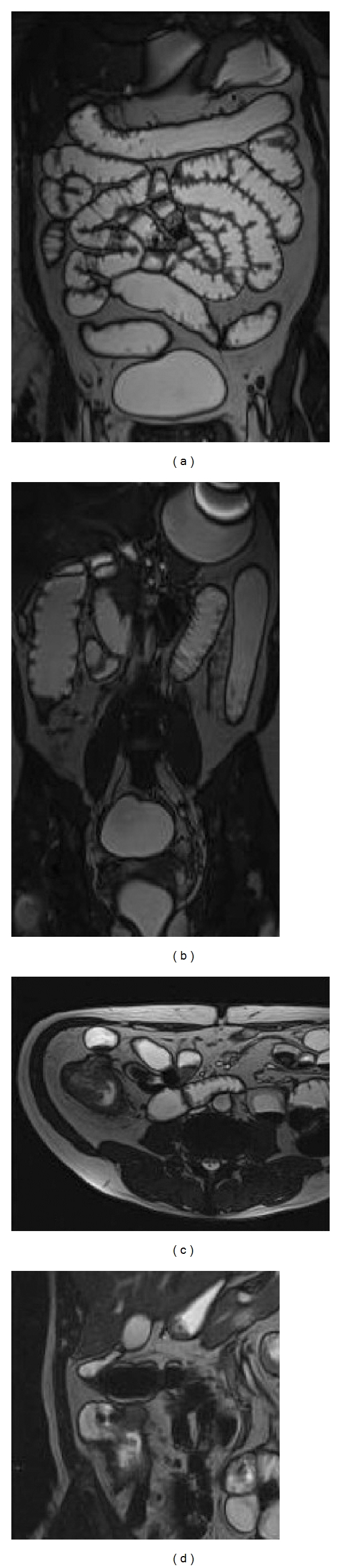
MR-E. Crohn's disease: colon involvement (a), (b) coronal TRUFI T2 showing (c) axial TRUFI T2; (d) coronal TRUFI T2.

**Figure 9:**
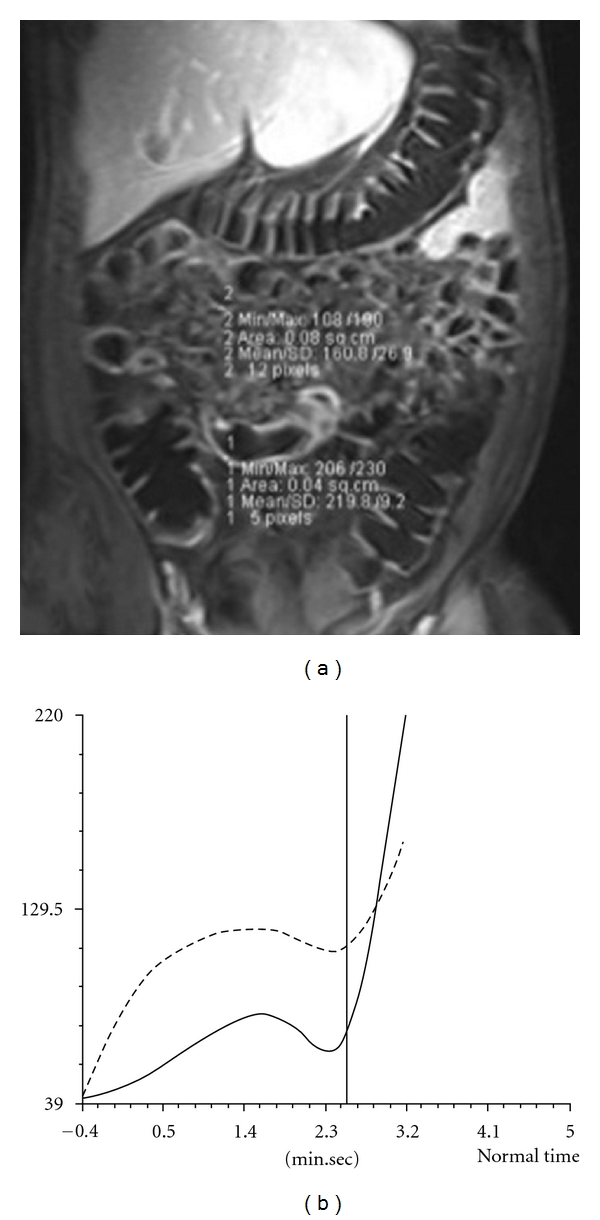
MR-E. (a) Coronal TRUFI T2 showing ileal loop wall thickening. (b) Intensity/time curve not showing inflammatory activity.

The study protocol includes unenhanced and enhanced scans (Figures 7 and 8) and the postcontrast acquisitions are processed, and the time-intensity (T/I) curves are evaluated (Figure 9).

MR enteroclysis—thanks to the adequate distension it provides all bowel loops, including the jejunum and the proximal small-bowel loops—is the technique that best enables accurate assessment of Crohn's disease (sens. 74%, spec. 91%) [7]. The technique, in fact, enables identification of lesions to the mucosa and complications. MR enteroclysis enables identification of a stenotic segment, evaluation of significance or insignificance, definition of characteristics—whether inflammatory or fibrous—and therefore guidance towards the most appropriate treatment. The possibility of obtaining dynamic-functional information, which can only be achieved with MR enteroclysis, makes it a unique imaging modality [61].

The correlation for the assessment of activity between endoscopy and MR and differentiation between mild and severe lesions is considered very high [7].

The main drawbacks are represented by high operating costs and the need for contrast injection. It also has relatively lower sensitivity than MDCT-E (60% versus 89% in the assessment of bowel-wall thickening only) [59].

However, MRI can detect the most relevant findings in patients with IBD with an accuracy superior to that of enteroclysis (sens. diagnosis 95.2%, abscesses 77.8%, fistulae 70.6%) [62]. 

To date, the elevated soft-tissue contrast and the functional information it can provide make MR imaging an ideal candidate for diagnosis at a young age and followup of patients with Crohn's disease [43, 50, 63].

## 7. 99mTc-HMPAO-Labeled Leukocyte Scintigraphy (TLLS)

99mTc-HMPAO-labeled leukocyte scintigraphy can identify the location of the inflammation and consequently assess its activity level.

Therefore, it provides rationale not only when clinical data and CT data are unclear but also during the assessment of a previously known inflammatory process so as to devise a suitable therapy program [64].

TLLS, used as a tool to identify location, level of inflammation, and level of infection in nuclear medicine can boast of a well-established tradition. The response to an inflammation or acute infection process is characterized by an increase in the local amount of blood, increased vascular permeability in addition to plasma protein exudation, and leukocyte stream [65].

During an acute inflammation/infection process, we witness a majority of polymorphic nuclear infiltrating cells while in chronic inflammation/infection cases cellular response mostly concerns lymphocytes, monocytes, and macrophages.

Although Crohn's disease represents a chronic condition with a predominant mononuclear infiltrate, labeled leukocytes, in the case of leukocytes infiltrating at the level of the mucosa, give operators a clear view of intestinal inflamed tracts during the acute phase of the disease [64].

TLLS has been extensively employed in patients affected by Crohns disease throughout the years producing not always unanimous results due to the differences among labeled cells, labeling methods, and the type of imaging which is obtained. Through the years, many labeling methods have been put forward both in vitro and in vivo. In vivo labeling is easy to perform and does not require special equipment. It reduces performing times as well as being well tolerated by patients because it calls for only one injection. Conversely, the generally low quality of view obtained by labeling methods which present great residual decreases the tracer substance amount needed for tracing diapedesis. As a result, sensitivity obtainable from this type of examination is lower because image quality is distinctly inferior.

In vivo labeling can be performed by means of technetium-99 m-labeled anti-granulocyte antibodies. The exam neither requires patient's preparation nor blood count, nor does it need subsequent separation of leukocyte components. 

In vitro leukocyte labeling, on the other hand, has the disadvantage of more complex procedures. It also demands trained staff, well-equipped laboratories for cell labeling in aseptic conditions not to mention longer performing times. The undeniable advantage of in vitro labeling is, however, given by its distinctly higher image quality due to a selective labeling of granulocytes with lower residual [65].

In our experience, 99mTc-HMPAO-labeled leukocyte scintigraphy (TLLS) is performed upon in vitro labeling. As for the whole-body 99mTc-HMPAO-labeled autologous leukocyte scintigraphy, a sample of venous blood of 50 mL is taken through syringes containing 10 mL of acid citrate dextrose formula A (ACD-A) upon adding 5 mL of hydroxyethyl starch (HES) at 10%. After sedimentation and centrifugation, a leukocyte button is obtained and then labeled with 740 MBq 99mTc-HMPAO (hexamethyl-propyleneamine oxime) (CERETEC GE, Healthcare) [38].

After labeling and quality control, the level of neutrophils in suspension is higher than 95%, and cellular activity reached 99%. Autologous leukocytes are injected intravenously. The actual dose, which is given according to the labeling result, ranges from 370 to 555 MBq.

Scintigraphy images are obtained by employing a rectangular dual-head large-field-of-view digital gamma camera equipped with high-resolution and low-energy collimators.

Images are obtained 60 and 180 m after labeled leukocytes have been administered again.

Compared to early scan, late scan (3 h) has a higher sensitivity (85% versus 100%) and accuracy (85% versus 95%) in identifying patients with active IBD and in defining IBD extension [66].

Still front/rear images of the abdomen and pelvis are acquired. In order to further assess perianal and rectal involvement, a projection of the pelvis is carried out in five cases, the aim being to separate such districts, which overlap during anterior projection, from the bladder. So as to have a clear view of the liver and transverse colon, the anterior projections of the standing patient's abdomen are acquired.

In the last few years, technetium-labeled hexamethyl-propyleneamine oxime scintigraphy (99mTc-HMPAO) has proved itself to be extremely useful in the diagnosis and followup to Crohn's disease. There are a great number of advantages to it, for instance, high sensitivity and specificity, low invasiveness, the possibility of performing the exam even in the acute phase of the disease, the opportunity to perform the exam even without bowel cleansing, the further opportunity to evaluate the seriousness of the process and multifocality at the same time, lack of risks or contraindications, no side effects, and good tolerance on the part of the patient.

However, procedures are long and complex not to mention the fact that they require blood manipulation and have low anatomic resolution. Moreover, 99mTc-HMPAO manages to visualize only acute inflammatory cases and may show false positives (*Yersinia* enteritis, ischemic colitis, tubercular enteritis, rejection, pseudomembranous colitis, vasculitis, appendicitis, hematomas, radiation enteritis, malignant pathologies with necrotic component). Crohn's disease case studies in nontreated patients report sensitivity percentage values of 83–98% and specificity percentage values of 92–100% [64, 65]; particularly in Crohn's disease diagnosis sensitivity percentage amounts to 90%, specificity to 93 [8]; in postsurgical recurrences, diagnostic accuracy percentage amounts to 81.3–81.8% [23].

**Figure 10:**
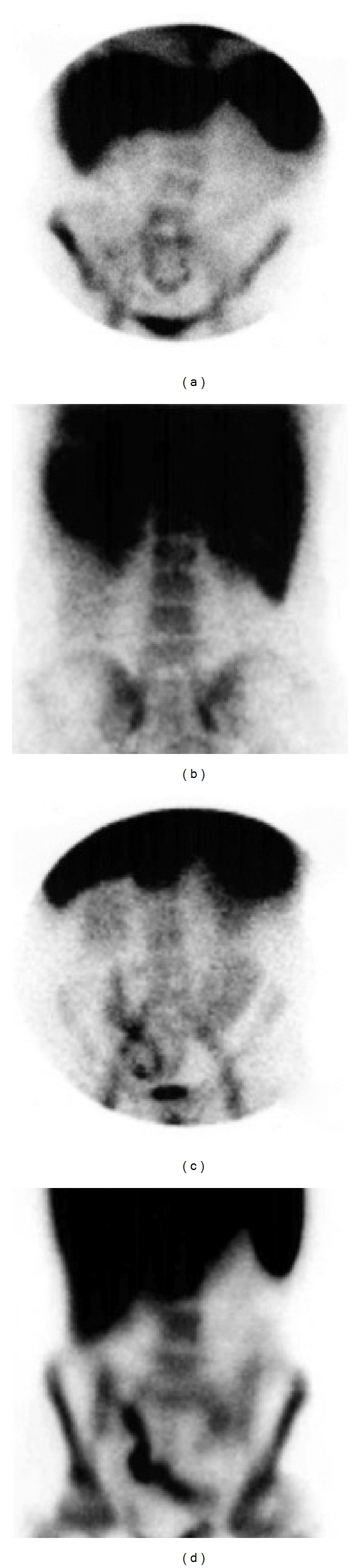
TLLS. (a) No inflammatory activity; (b) mild inflammatory activity; (c) moderate inflammatory activity; (d) severe inflammatory activity.

To date, the existence of (almost) standard criteria (assessment of light activity, be it moderate or serious depending upon the level of the uptake of the bone marrow at the level of the ileac crests) make it possible for the exam to be reproduced and consequently be valid for identifying the degree of inflammation (Figure 10).

## 8. Conclusion

In recent years, several radiologic techniques have been developed for the study of the small bowel. Each technique is characterized by its own profile of advantages and disadvantages (Table 1).

Because of the relapsing nature of Crohn's disease and the young age at which it usually develops, frequent reevaluation of disease is necessary in many patients.

Specific, noninvasive, well-tolerated, and inexpensive examinations should be carried out while studying Crohn's disease. These examinations will have to confirm clinical suspicion of the disease as well as provide morphological information such as location, extension, or complication and recurrence evolution (Table 2).

Examinations should also make functional information available for an effective management of the disease.

In our experience, in the case of initial diagnosis, any investigation can be used, considering the experience of the structure and the operator; we propose MDCT, MRE, or CE for the first diagnosis; US, and possibly supplemented with PD/CEUS, for followup; MRE, MDCT, or TLLS for Relapses; MDCT, MRE, or CE for complications (Table 3) [52].

## Figures and Tables

**Table 1:** Imaging techniques features in Crohn's disease.

	Invasiveness	IV infusion	Nasoenteric tube	Well accepted	Widely available	Operator dependent	Radiation exposure	Superficial lesion detection	Intra/ extraintestinal structures	Activity of disease
CE	+	−	+	−	+	−	+	+	−	−
US	−	−	−	+	+	+	−	−	−	−
PD	−	−	−	+	+	+	−	−	−	−
CEUS	−	+	−	+	−	+	−	−	−	+
MDCT-E	+	+	+	−	−	−	+	−	+	+/−
MR-E	+	+	+	−	−	−	−	−	+	+/−
TLLS	−	+	−	−	−	−	+	−	−	+

CE: conventional enteroclysis; US: ultrasonography; PD: power Doppler; CEUS: contrast-enhanced ultrasonography; MDCT-E: multidetector CT enteroclysis; MR-E: magnetic resonance enteroclysis; TLLS: 99mTc-HMPAO-labeled leukocyte scintigraphy; IV infusion: intravascular infusion.

**Table 2:** Diagnostic accuracy of CE, US, CEUS, CT, MR, and TLLS in the assessment of diagnosis, disease activity, complication and, relapses in Crohn's disease.

		CE			US			CEUS		CT			MR enteroclysis		MR enterography		TLLS		
		Sens.	Spec.	Acc.	Sens.	Spec.	Acc.	Sens.	Spec.	Sens.	Spec.	Acc.	Sens.	Spec.	Sens.	Spec.	Sens.	Spec.	Acc.
Diagnosis	*Panes*				85	98							78	85					
	*Horsthius*				90	96				84	95		93	93					
	*Fraquelli*				75	94													
	*Alberini*																83–98	92–100	
	*Rispo *	98	97		92	97											90	93	
	*Rieber*	85	77										95	93					
	*Lee*														83	100			

Disease activity	*Migaleddu 2009*							93	94										
	*Serra*							81	63										
	*Lee 2009*									89	90								
	*Panes*									81	88								

Complication	*Panes*									81	98								
Fistulae	*Maconi *	70	95										71						
	*Rieber*	18																	
Abscesses	*Maconi *			80								77							
	*Rieber*	0											78						
	*Panes*						87					92							
Stenosis	*Cappabianca*												90	100	89	67			
Extraintestinal	*Lee*														100				

Relapses	*Tarjàn*	88	93																
	*Paredes*																		78

CE: conventional enteroclysis; US: ultrasonography; PD: power Doppler; CEUS: contrast-enhancement ultrasonography; MDCT-E: multidetector CT enteroclysis; MR-E: magnetic resonance enteroclysis; TLLS: 99mTc-HMPAO-labeled leukocyte scintigraphy; IV infusion: intravascular infusion; sens.: sensitivity; spec.: specificity; acc.: accuracy.

**Table 3:** Appropriateness of examination in Crohn's disease.

	CE	US	CPD/CEUS	MDCT-E	MDCT-e	MR-E	MR-e	TLLS
First diagnosis	8	7	7	9	8	9	8	7
Followup*	2	9	6	2	2	4	5	2
Relapse	6	6	6	8	7	9	8	7
Complications	7	6	6	9	8	9	8	6

9: extremely appropriate; 7-8: usually appropriate; 4–6 doubt; 2-3: usually inappropriate; 1: extremely inappropriate [52].

CE: conventional enteroclysis; US: ultrasonography; PD: power Doppler; CEUS: contrast-enhancement ultrasonography; MDCT-E: multidetector CT enteroclysis; MDCT-e: multidetector CT enterography; MR-E: magnetic resonance enteroclysis; MR-e: magnetic resonance enterography; TLLS: 99m Tc-HMPAO-labeled leukocyte scintigraphy; IV infusion: intravascular infusion; *negativity of clinic and laboratory exams.
